# Evaluation of Choroidal Thickness in Patients with Pseudoexfoliation Syndrome and Pseudoexfoliation Glaucoma

**DOI:** 10.1155/2016/3545180

**Published:** 2016-06-14

**Authors:** Ayhan Dursun, Ayse Vural Ozec, Oznur Dogan, Feyza Gulac Dursun, Mustafa Ilker Toker, Aysen Topalkara, Mustafa Kemal Arici, Haydar Erdogan

**Affiliations:** ^1^Department of Ophthalmology, Cumhuriyet University, School of Medicine, 58140 Sivas, Turkey; ^2^Department of Ophthalmology, Numune Hospital, 58000 Sivas, Turkey

## Abstract

*Purpose*. To compare the macular and peripapillary choroidal thickness in eyes with pseudoexfoliation (PEX) syndrome and PEX glaucoma with the normal eyes of healthy controls.* Materials and Methods*. In this prospective study, 30 eyes of 30 patients with PEX syndrome, 28 eyes of 28 patients with PEX glaucoma, and 30 eyes of 30 age-matched healthy subjects were enrolled. Choroidal thicknesses in the macular and peripapillary areas were measured by using spectral domain optical coherence tomography.* Results*. Gender, age, and axial length did not significantly differ between the groups (all, *p* > 0.05). The mean values of choroidal thickness in the macular and peripapillary areas (except the superior quadrant) in the patients with PEX syndrome and PEX glaucoma were lower compared with controls (all *p* < 0.05). The mean values of the macular and peripapillary choroidal thickness in the PEX glaucoma group were lower compared with PEX syndrome group; however this difference was not significant.* Conclusions*. The findings of this study revealed that macular and peripapillary choroidal thicknesses were decreased in PEX syndrome and PEX glaucoma cases. The role of choroid in the development of glaucomatous damage in patients with PEX syndrome remains unclear.

## 1. Introduction

Pseudoexfoliation (PEX) syndrome and PEX glaucoma are age-related disorders characterized with the production and progressive deposition of extracellular fibrillary material in various ocular tissues [1]. PEX glaucoma develops as a result of blockage in the trabecular meshwork formed by the pseudoexfoliative material and pigment, and globally is the most common form of secondary open-angle glaucoma [2, 3]. Pseudoexfoliative material affects the structures in the anterior ocular segment, like the corneal endothelium, surface of the lens, and trabecular meshwork; however it has been shown that it may also affect the structures in the posterior ocular segment, like the posterior ciliary arteries (PCAs), vortex veins, and central retinal vessels [4, 5].

In recent studies, evaluating retrobulbar blood flow in patients with PEX syndrome and PEX glaucoma, it was shown that the hemodynamic parameters of the ophthalmic artery were affected in both situations [6, 7]. Choroid tissue has one of the highest rates of blood flow in the human body and vascular supply in this tissue is derived primarily from the long and short ciliary arteries with some contribution from the anterior ciliary arteries [8]. Measurement of the choroidal thickness can give us important information about the choroidal blood flow rate. With the application of spectral optical coherence tomography (SD-OCT) systems, it is possible to capture the image of the full-thickness of the choroid in vivo [9]. Goktas et al. have found that the macular choroidal thickness in patients with pseudoexfoliation syndrome is lower than that of healthy individuals [10]. In a study evaluating macular choroidal thickness in the patients with PEX glaucoma, Bayhan et al. found the choroidal thickness in the nasal region to be thinner compared with the control group [11]. In another study, it was reported that glaucoma has developed in about 32% of patients with unilateral PEX syndrome who were followed up for ten years [12]. However the pathophysiology of conversion from PEX syndrome to PEX glaucoma has not been completely understood.

The peripapillary area and optic disc are important in patients with glaucoma. The peripapillary choroid plays an important role in supplying blood to the optic nerve head [13]. Evaluation of the peripapillary choroid is very important, since glaucomatous optic neuropathy is believed to occur at the optic nerve head [14]. In this study, we conducted measurements of macular and peripapillary choroidal thickness using cross-sectional images of SD-OCT in normal control, PEX syndrome, and PEX glaucoma subjects. The objective of this study was to find out the difference of choroidal thickness and related factors between the PEX syndrome and PEX glaucoma.

## 2. Materials and Methods

This prospective study includes 30 eyes of 30 patients with PEX syndrome, 28 eyes of 28 patients with PEX glaucoma, and 30 eyes of 30 healthy subjects. The participants were recruited from the Ophthalmology Department of the Cumhuriyet University between June 2014 and March 2015. The study was approved by the Cumhuriyet University Institutional Review Board and Ethics Committee and adhered to the tenets of the Declaration of Helsinki. All the participants provided written informed consent prior to the study.

All subjects underwent full ophthalmic examinations, including the best-corrected visual acuity (BCVA), refraction, slit-lamp biomicroscopy, intraocular pressure with Goldmann applanation tonometer, gonioscopy, binocular optic disc and retina evaluation, and fundus photography. Axial length was measured using ultrasonic biometry (NIDEK US-4000 Echoscan, Gamagori, Japan), and visual field examination was made using the Humphrey perimetry (Carl Zeiss Meditec, Dublin, CA) with the 30-2 program. The presence of clinical PEX syndrome was defined as having PEX material deposits on the edge of pupil and/or the lens capsule, an IOP of less than 21 mmHg, normal optic disc, and normal visual field findings. PEX glaucoma diagnosis was made in the presence of PEX material on the anterior lens capsule or edge of pupil and/or in the angle on gonioscopy, an intraocular pressure more than 21 mmHg, glaucomatous optic nerve damage, and change in visual field [15]. A glaucomatous visual field change was defined as four abnormal points with *p* < 5% on the pattern deviation plot, both confirmed at least once or a glaucoma hemifield test result, outside normal limits [15]. Healthy subjects who attended our clinic with findings of IOP ≤ 21 mmHg, a normal appearance of the optic nerve head, and normal visual field test results were included the study.

Patients with chronic systemic diseases like diabetes mellitus or arterial hypertension were not included the study. Moreover, patients below 18 years of age and who showed the following characteristics were excluded from the study: a BCVA < 20/40, a spherical refraction outside ±5.0 diopters or cylinder correction outside ±3.0 diopters, anamnesis of ocular trauma or surgery, an inflammatory eye disease, and retinal diseases like diabetic retinopathy and macular degeneration.

### 2.1. Image Acquisition and Image Processing Procedures

**Figure 1:**
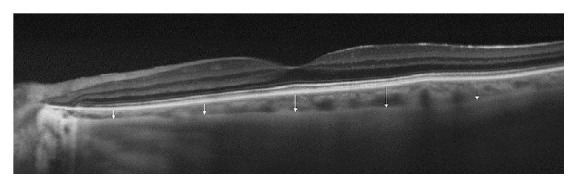
Optical coherence tomography scan, showing the macular choroidal thicknesses at five different locations (subfoveal, 1.5 mm nasal to the fovea, 3 mm nasal to the fovea, 1.5 mm temporal to the fovea, and 3 mm temporal to the fovea).

**Figure 2:**
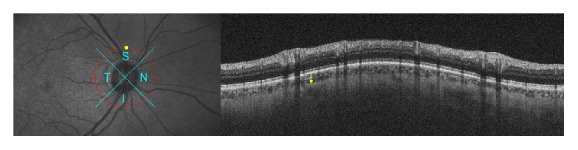
Measurement of peripapillary choroidal thickness patient with PEX glaucoma. Yellow arrow indicates the choroidal thickness in the superior quadrant.

Choroidal thickness measurements were conducted by the same experienced technician using the RS-3000 Advance OCT Retina Scan (NIDEK, Gamagori, Japan), which is a high-speed SD-OCT/confocal ophthalmoscope system. In the macula, 12 radial scans, 9 mm length, were centered onto the fovea, by using choroidal mode. Choroidal thickness was measured manually and perpendicularly from the outer edge of the hyperreflective retina pigment epithelium to the choroid-sclera boundary at the fovea, and 1.5 mm nasal, 3 mm nasal, 1.5 mm temporal, and 3 mm temporal to the fovea (Figure 1). In order to evaluate the peripapillary choroidal thickness, images were taken at the disc circle mode. The disc circle scan pattern is an image of circle in 3.45 mm diameter centering on the disc. In these scans, the peripapillary choroidal thickness was measured as defined above, in the superior, inferior, temporal, and nasal quadrants, using the producer's software NAVIS-EX 1.3.6 (Nidek Advanced Vision Information System, NIDEK Co. Ltd.) (Figure 2). For elimination of diurnal variations, the choroidal thickness has been measured between 10 and 12 a.m. All the SD-OCT measurements were obtained by the same clinician (AD) and a signal strength index greater than 6/10 (maximum) was included.

### 2.2. Statistical Analysis

Statistical analysis was performed using a commercially available statistical software package (version 16.0; SPSS Inc., Chicago). The descriptive statistics were expressed as the mean ± standard deviation. The differences in choroidal thickness between patients in the groups were analyzed by the independent-samples *t*-test. A *p* value less than 0.05 was considered statistically significant.

## 3. Results

The demographic data and clinical characteristics of subjects by groups are summarized in Table 1. Age, sex, axial length, spherical equivalent, and IOP values were similar in the three groups (all, *p* > 0.05). The average mean deviation was significantly different between the PEX glaucoma and other two groups (*p* < 0.001).

The mean subfoveal choroidal thickness measurements at each location are shown in Table 2. Choroidal thickness measurements at all locations were significantly lower in the patients with PEX glaucoma and PEX syndrome, compared to control group (both, *p* < 0.05). When compared with the PEX syndrome group, the mean choroidal thicknesses were thinner in the PEX glaucoma group; however, this difference was not statistically significant. The mean peripapillary choroidal thickness measurements at each location are shown in Table 2.

Apart from the superior quadrant, choroidal thickness measurements at all the peripapillary locations were significantly lower in the patients with PEX glaucoma and PEX syndrome, compared with the control group (both, *p* < 0.05). In the superior quadrant, the difference was significant only between the patients with PEX glaucoma and control group (*p* = 0.013). When compared with the group with PEX syndrome, the mean peripapillary choroidal thicknesses were thinner in the PEX glaucoma group but this difference was not statistically significant.

## 4. Discussion

The pathophysiology of PEX syndrome and PEX glaucoma is not completely known. The presence of pseudoexfoliative material has been shown in the vessels supplying both the anterior and posterior segments of the eye [5]. PEX glaucoma occurs in 5.3% of cases with PEX syndrome within five years and in 15.4% of them within ten years [16], and, besides this, PEX glaucoma progresses relatively more rapidly and may be resistant to medical treatment; it is therefore important to anticipate that PEX glaucoma may develop in the patients with PEX syndrome [17].

To the best of our knowledge, there is no study in the literature that compares the macular and peripapillary choroidal thicknesses between the patients with PEX syndrome and PEX glaucoma. In the present study, both macular and peripapillary choroidal thicknesses were found to be thinner in the patients with PEX syndrome and PEX glaucoma, compared with the controls. The choroid was thinner in the eyes of patients with PEX glaucoma compared with PEX syndrome, but this difference was not statistically significant.

There is a great number of studies demonstrating that parameters of retrobulbar blood flow change in the PEX syndrome [6, 7, 15]. Dayanir et al. have found that values of ophthalmic artery peak systolic velocity (PSV) and end-diastolic velocity (EDV) were lower in patients with unilateral PEX syndrome, compared with control group [7]. In recent years, measurements made of choroidal thickness using OCT may reveal useful information for the evaluation of retrobulbar blood flow. In the 2011 Beijing Eye Study, You et al. did not determine a relationship between the PEX syndrome and choroidal thickness [18]. Contrary to this, Goktas et al. determined choroidal thickness in PEX syndrome and determined a thinner choroid in the subfoveal, temporal, and nasal quadrants, compared with controls [10]. They have considered that the thinner choroid in PEX syndrome might be due to the increased vascular resistance and decreased blood flow. The choroid was also found to be thinner in our study in the macular and peripapillary areas (except the superior quadrant) in the PEX syndrome, compared with the control group. Small vessels are affected in PEX syndrome rather than large vessels, and these pathological changes were considered to cause a thinner choroid in PEX syndrome [4, 19–21].

There are various studies investigating whether retrobulbar hemodynamics change in PEX glaucoma [6, 15, 22]. Galassi et al. determined that retrobulbar hemodynamics were impaired in the patients with PEX glaucoma, compared with cases of primary open-angle glaucoma and healthy controls [23]. They claimed the presence of impaired ocular vascular regulation in PEX glaucoma. Bayhan et al. determined a thinner choroidal thickness in the nasal quadrant in patients with PEX glaucoma, compared with a control group of healthy individuals [11]. Similar to the results of previous studies indicating that retrobulbar hemodynamics are affected by PEX glaucoma, we also determined a thinner choroidal thickness in the macular and peripapillary regions, compared with healthy subjects. Unlike the results of Bayhan et al., the choroidal thickness in our study was found to be thinner in all quadrants, including the nasal quadrant, compared with the controls. This difference may arise from the relatively small number of cases, the measurement of choroidal thickness by using manual segmentation techniques in both studies, and the different patient profiles.

PEX syndrome may lead to PEX glaucoma [24]. In some studies, the importance of the high values of intraocular pressure (IOP) and the presence of a greater diurnal variation have been shown in the presence and pathogenesis of PEX glaucoma [13, 25, 26]. However, Detorakis et al. found EDV scores to be significantly lower and resistivity index scores significantly higher at short posterior ciliary artery (SPCA) in exfoliation glaucoma, compared with nonglaucomatous exfoliative eyes, and they considered that PEX glaucoma might be associated with hemodynamic impairment at SPCA [6]. In the present study, we investigated whether impaired choroidal blood flow played any role in the conversion from PEX syndrome to PEX glaucoma and determined a thinner choroidal thickness in PEX glaucoma, compared with PEX syndrome. However this difference was not statistically significant.

Jonas et al. determined that choroidal thicknesses in foveal and parafoveal regions, in patients with primary open-angle glaucoma, were similar to those in healthy subjects [27]. Similarly, Hosseini et al. found similar choroidal thicknesses in peripapillary and macular regions of patients with POAG and control subjects [28]. Studies showing thinner choroidal thicknesses in the PEX syndrome and PEX glaucoma lead us to consider that the exfoliative material may impair the choroidal circulation, by affecting the vessels in retrobulbar area [10, 11]. Nevertheless, in our study, PEX syndrome and PEX glaucoma did not differ with regard to choroidal thickness, and this result leads us to suggest that some possible factors, other than the choroidal blood flow, may play a role in the conversion from PEX syndrome to PEX glaucoma.

This study has some potential limitations. The first limitation was the relatively small number of patients in the study groups. Our study included precise inclusion criteria, like the exclusion of cases with cataract from the study, in order to maintain the similarities of age, gender, and axial length in the control and patient groups and to accurately measure choroidal thickness. The second limitation was the measurement of choroidal thickness with OCT as a whole, and the retinal pigment epithelium and choriocapillaris, which are important for the blood supply of photoreceptors, could not be measured separately. The third limitation was the manual measurement of choroidal thickness, due to the absence of automatic segmentation software. The fourth limitation was the use of one or more kinds of antiglaucoma agents by all of the patients with PEX glaucoma. There are studies in the literature showing that antiglaucoma agents affect the choroidal thickness, but controversial results also exist [29–32]. The choroidal thicknesses of those cases with PEX glaucoma might be affected by these medications. Our fifth limitation is the measurement of the choroidal thickness only by one clinician. Although we have not included the patients with systemic hypertension history, the unassessment of systolic and diastolic blood pressure and the ocular perfusion pressure that can affect the choroidal thickness is another limitation.

## 5. Conclusion

In conclusion, the macular and peripapillary choroidal thicknesses in this study were found to be thinner in the subjects with PEX syndrome and PEX glaucoma, compared with the control group. Choroidal thicknesses in the eyes with PEX glaucoma were thinner compared with PEX syndrome, but the difference was not statistically significant. Further studies are needed to investigate the effect of change in choroidal thickness on the development of glaucoma in the cases with PEX syndrome.

## Figures and Tables

**Table 1:** Demographic and clinical characteristics of patients by groups.

Variable	Control	PEX syndrome	PEX glaucoma	*p*
Age (y)	68.3 ± 7.2	70.4 ± 4.3	70.8 ± 8.1	0.434
Sex (female/male)	6/24	8/22	6/22	0.664
AXL (mm)	23.2 ± 0.8	23.3 ± 0.9	22.9 ± 0.8	0.173
SE (D)	1.4 ± 1.1	1.0 ± 0.6	1.3 ± 1.0	0.622
MD (dB)	−0.45 ± 0.3	−0.58 ± 0.2	−7.7 ± 6.7	<0.001
IOP (mmHg)	13.5 ± 2.1	14.9 ± 3.6	13.3 ± 3.4	0.095

PEX: pseudoexfoliation; AXL: axial length; SE: spherical equivalent; MD: mean deviation; IOP: intraocular pressure.

**Table 2:** Mean thicknesses of the macular and peripapillary choroid in each group.

	Control	PEX syndrome	PEX glaucoma	*p*1	*p*2	*p*3
*Macular choroidal thickness*						
Subfoveal	280.10 ± 63.83	223.96 ± 81.51	216.03 ± 93.31	0.004	0.003	0.727
1.5 mm nasal to fovea	242.13 ± 75.32	195.46 ± 75.06	180.86 ± 66.23	0.019	0.001	0.428
3.0 mm nasal to fovea	162.00 ± 50.83	124.83 ± 62.90	106.80 ± 48.49	0.015	<0.001	0.219
1.5 mm temporal to fovea	258.26 ± 48.15	203.76 ± 70.10	201.80 ± 77.39	0.001	0.001	0.918
3.0 mm temporal to fovea	239.56 ± 46.68	181.96 ± 62.62	187.23 ± 58.70	<0.001	<0.001	0.738

*Peripapillary choroidal thickness*						
Temporal	156.56 ± 40.33	128.75 ± 58.31	122.53 ± 32.40	0.039	0.001	0.616
Nasal	156.43 ± 45.53	130.36 ± 47.28	121.26 ± 45.87	0.034	0.004	0.452
Superior	165.40 ± 43.23	146.10 ± 59.83	137.00 ± 43.00	0.160	0.013	0.504
Inferior	147.96 ± 46.13	121.10 ± 38.11	113.80 ± 27.52	0.017	0.001	0.399
Average	156.59 ± 33.84	131.33 ± 46.82	123.65 ± 33.22	0.022	<0.001	0.472

PEX*: pseudoexfoliation; p*1*: *comparison between control and PEX syndrome groups by Student's *t*-test; *p*2*: *comparison between control and PEX glaucoma groups by Student's *t*-test; *p*3*: *comparison between PEX syndrome and PEX glaucoma groups by Student's *t*-test.
